# Background removal for debiasing computer-aided cytological diagnosis

**DOI:** 10.1007/s11548-024-03169-0

**Published:** 2024-06-25

**Authors:** Keita Takeda, Tomoya Sakai, Eiji Mitate

**Affiliations:** 1 School of Information and Data Sciences, Nagasaki University, 1-14 Bunkyo, Nagasaki, 8528521 Japan; 2 Department of Oral and Maxillofacial Surgery, Kanazawa Medical University, 1-1 Daigaku, Uchinada, Kahoku, Ishikawa 9200293 Japan; 3 Graduate School of Integrated Science and Technology, Nagasaki University, 1-14 Bunkyo, Nagasaki, 8528521 Japan

**Keywords:** Deep learning, Robust principal component analysis, U-Net, Data cleaning, Oral cytology

## Abstract

To address the background-bias problem in computer-aided cytology caused by microscopic slide deterioration, this article proposes a deep learning approach for cell segmentation and background removal without requiring cell annotation. A U-Net-based model was trained to separate cells from the background in an unsupervised manner by leveraging the redundancy of the background and the sparsity of cells in liquid-based cytology (LBC) images. The experimental results demonstrate that the U-Net-based model trained on a small set of cytology images can exclude background features and accurately segment cells. This capability is beneficial for debiasing in the detection and classification of the cells of interest in oral LBC. Slide deterioration can significantly affect deep learning-based cell classification. Our proposed method effectively removes background features at no cost of cell annotation, thereby enabling accurate cytological diagnosis through the deep learning of microscopic slide images.

## Introduction

Cytological diagnosis involves microscopic examination of cells obtained through either exfoliation or intervention. The most common targets of cytology are cervical cells, breast cancers, and respiratory organs [18]. As the accuracy of diagnosing cellular atypia depends on the expertise of pathologists, automated cytological diagnosis based on image processing is considered more effective and reproducible. Deep learning techniques, specifically convolutional neural networks (CNNs), have emerged as popular data-driven approaches for image-based cytology. Weakly supervised object localization [20, 30] has enabled automatic object detection and classification without expensive manual annotations for each object [13, 26]. By providing a label indicating the presence of malignant cells in each whole-slide image for training, a CNN with class activation mapping (CAM) [15, 30] can be trained to classify the images while highlighting the cells of interest, even without information on the location and malignancy of each cell.

However, slide images with different acquisition or storage conditions may contain latent image features that can spuriously be correlated with cell characteristics. The causes and details of slide deterioration remain unclear, but it is known that microscopic slides can turn yellow or brown over time [2, 19]. Prior storage and imaging of microscopic slides did not necessarily assume the future use of these images as a dataset for machine learning to automatically identify discriminative cell features. There is a possibility of a false correlation between the cell features and whole-slide image features, if the facilities that collected and stored the specimens or the operators who digitized the slides had biases toward benign or malignant types.

**Fig. 1:**
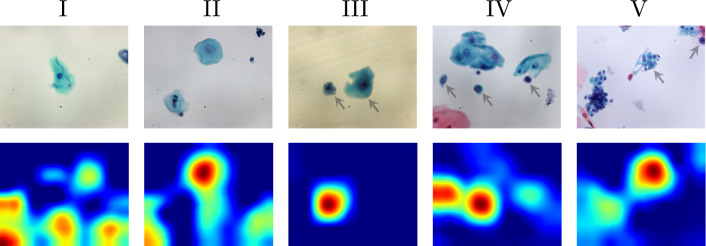
Examples of oral cells and Grad-CAM. The first row gives microscopic slide images of Pap classes I–V. The second row presents the corresponding Grad-CAMs obtained from a transfer learning classifier

To illustrate the problems that non-cellular image information poses for deep learning, we introduce oral cytology as a specific example. The first row of Fig. 1 shows examples of cell slide images used for non-invasive diagnosis of oral cancer.^1^ Epithelial cells from the oral cavity were collected by scraping with a cotton swab or special brush. ThinPrep Hologic’s tools for liquid-based cytology (LBC) [11] produce slides with less cell overlap and debris. The Papanicolaou-stained cell slides were photographed with a 40x objective lens and stored as raster images. Papanicolaou (Pap) staining [21] is a common cytopathological staining technique used to differentiate cells in smear preparations. Medical experts visually inspected each image and assigned one of the so-called Pap class labels, I to V. Pap classes I and II are considered normal, whereas Pap classes III and above are suspected to be abnormal. No information is available regarding the position, orientation, and background of the cells with respect to their class. Medical experts classify the specimens based on the ratio of the size of the entire cell to that of its nucleus, the relationship between the size and roundness of the cell nucleus, and whether the Pap-stained cells are blue in color.

The dataset of cytology images shown in Fig. 1 has two underlying causes that prevent a deep learning classifier from learning appropriate image features.

- The image background provides information about subject. Notably, the background color and pattern vary across images. This is because the images are selectively captured from a wide slide of a subject and the background is dependent on the subject. In cases where the dataset lacks sufficient subjects or where slide deterioration is indicative of cell type, a CNN classifier can infer class identity by analyzing the distinctive features found in the background patterns.
- The ease of identifying classes based on individual cell features differs among classes. While Pap classes III–V are typically identified based on the presence of specific cells with abnormal features (indicated by the arrows in the first row of Fig. 1), Pap classes I and II are determined simply because none of the cells exhibit abnormal features, rather than based on specific cells as evidence.

Consequently, the classifier trained on the oral LBC image dataset may rely on background features. For instance, a transfer learning classifier fine-tuned from VGG16 [25] that was pre-trained on ImageNet [6], following a similar approach to that of Teramoto et al. [26], may focus on features beyond cells, as suggested by Grad-CAM [24] in the second row of Fig. 1. For Pap classes IV and V, the Grad-CAM tends to highlight the cell of interest marked by arrows to the first row of Fig. 1. However, the classifier appears to prioritize the background features for Pap classes I and II. Owing to the difficulty of identifying distinguishing cells in images of Pap classes I and II, there is a greater risk of inappropriately referring to background features.

To ensure impartial cell classification, it is essential to eliminate latent image features that are spuriously correlated with the cell classes. Merely masking the background is not sufficient because spurious features caused by slide deterioration can be present throughout the entire slide image. Using pixel annotation to mask the background is expensive, less effective, and impractical.

The contributions of this article are as follows:

- Substantiation of the influence of slide deterioration on deep learning-based cytology.
- Removing background bias in cytology images.
- Accurate semantic cell segmentation achieved with a small training dataset without segmentation annotation.

## Unsupervised deep learning for cell and background separation

**Fig. 2:**
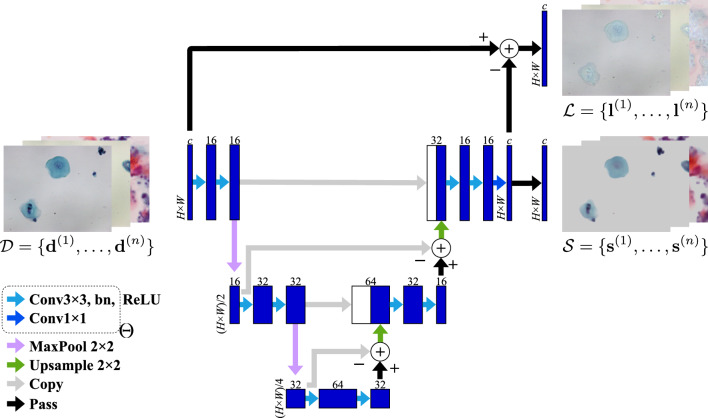
Network architecture for separating cells from background. The trainable parameters, $\Theta$, are optimized on training images, $\mathcal {D}$, so that the predicted cell images, $\mathcal {S}$, have as few nonzero connected pixels as possible and the predicted background images, $\mathcal {L}$, are as redundant as possible. Note that the $\textbf{s}^{(j)}$ is visualized with a constant added to the pixel value

### Model architecture

We designed a deep neural network model that encodes and decodes foreground cells to separate them from the background of an input cytology image. The model architecture is shown in Fig. 2. Let $\{\textbf{d}^{(j)}\}$ ($j=1,\dots ,n$) be a batch of *n* input cytology images, each of size $H\times W$, where $\textbf{d}^{(j)}\in \mathbb {R}^{c\times m}$ is a matrix storing *c* channels and $m=HW$ pixels of the *j*-th image. When the input image is grayscale, $c=1$, and when it is a color image, $c=3$. Our model takes $\textbf{d}^{(j)}$ as input, processes it as an $H\times W$ image, and outputs the corresponding image of cells as $\textbf{s}^{(j)}\in \mathbb {R}^{c\times m}$ together with the background $\textbf{l}^{(j)}=\textbf{d}^{(j)}-\textbf{s}^{(j)}\in \mathbb {R}^{c\times m}$. The nonzero pixels of $\textbf{s}^{(j)}$ indicate the cell regions.

We employed a dual-frame U-Net [12] with a set $\Theta$ of trainable parameters for encoding and decoding convolutional image features to produce $\textbf{s}^{(j)}$. The set $\Theta$ consists of convolutional filter kernels and scaling and shifting parameters. This improved U-Net model is suitable for learning foreground cell features because its unique structure incorporates the subtraction of encoder and decoder feature maps to balance coarse and fine image features.

### Loss function for unsupervised training

Suppose that a collection of cytology images has a low-rank and sparse nature [4]. That is, image backgrounds are redundant and can be synthesized with their principal components. In contrast, cells are sparse. A small number of connected pixels form the cell regions.

To train our model in Fig. 2, we introduce a loss function that exploits the low-rank and sparse nature of cytology images. For a batch $\{\textbf{d}^{(j)}\}$ of *n* training images, we obtain a couple of tensors $\mathcal {L}, \mathcal {S}\in \mathbb {R}^{n\times c\times m}$ with the corresponding batches $\{\textbf{l}^{(j)}\}$ and $\{\textbf{s}^{(j)}\}$ of *n* model outputs. We then unfold the tensors $\mathcal {L}$ and $\mathcal {S}$, respectively, into matrices $\mathcal {L}_{(1)}\in \mathbb {R}^{cm\times n}$ and $\mathcal {S}_{(2)}^\top \in \mathbb {R}^{c\times mn}$. To enhance the redundancy, sparsity, and pixel connectedness, we define the following loss function:

1 $$\begin{aligned} \Vert \mathcal {L}_{(1)}\Vert _* + \lambda \Vert \mathcal {S}_{(2)}^\top \Vert _{2,1} + \lambda _{\text{ TV }} \sum _{j=1}^{n}\sum _{k=1}^{c}\mathop {\text {TV}}\nolimits (\mathop {\text {mat}}\nolimits _{(H,W)}\mathcal {S}_{(3)}^{\top (jk)}). \nonumber \\ \end{aligned}$$

Here, $\Vert \mathcal {L}_{(1)}\Vert _*$ denotes the nuclear norm of $\mathcal {L}_{(1)}$ and is defined as the sum of the singular values of $\mathcal {L}_{(1)}$. The $\ell _{2,1}$ norm of $\mathcal {S}_{(2)}^\top$, denoted by $\Vert \mathcal {S}_{(2)}^\top \Vert _{2,1}$, is the sum of $\ell _2$ norms of pixelwise *c*-vectors. We refer to $\mathop {\text {TV}}\nolimits$ as an anisotropic approximation of total variation [5, 23]. For a single-channel image $\varvec{S}\in \mathbb {R}^{H\times W}$, the anisotropic total variation is defined as

2 $$\begin{aligned} \mathop {\text {TV}}\nolimits (\varvec{S}) = \sum _{h=1}^{H-1}\sum _{w=1}^{W-1} ( |S_{h+1,w}-S_{h,w}|+ |S_{h,w+1}-S_{h,w}|) \nonumber \\ \end{aligned}$$

where $S_{h,w}$ represents the pixel value of $\varvec{S}$ at (*h*, *w*). In Eq. (1), $\mathop {\text {mat}}\nolimits _{(H,W)}\mathcal {S}_{(3)}^{\top (i)}$ denotes an operator that reshapes the *i*-th column *m*-vector $\mathcal {S}_{(3)}^{\top (i)}\in \mathbb {R}^{m}$ ($i=1,\dots ,nc$) into a single-channel image of size $H\times W$. We take the sum of the anisotropic total variation for all channels for all images in $\mathcal {S}$.

We train our model by minimizing the loss function in Eq. (1) with respect to the trainable parameters $\Theta$. Minimizing the nuclear norm $\Vert \mathcal {L}_{(1)}\Vert _*$ and the $\ell _{2,1}$ norm $\Vert \mathcal {S}_{(2)}^\top \Vert _{2,1}$ encourages the background to be low-rank and cell images to be sparse. Minimizing the total variation promotes the number of pixels in $\mathcal {S}$ corresponding to edges to be small. The two nonnegative hyperparameters, $\lambda$ and $\lambda _{\text{ TV }}$, balance each member of the loss. When the input is a grayscale image ($c=1$), $\Vert \mathcal {S}_{(2)}^\top \Vert _{2,1}$ can be simplified as $\Vert \mathcal {S}_{(2)}^\top \Vert _1$. When $c=1$ and $\lambda _{\text{ TV }}=0$, as a special case, our loss function is further simplified as

3 $$\begin{aligned} \Vert \mathcal {L}_{(1)}\Vert _* + \lambda \Vert \mathcal {S}_{(2)}^\top \Vert _{1}, \end{aligned}$$

which is equivalent to the objective function of the robust principal component analysis (RPCA) [4]. It is theoretically reasonable to choose $\lambda =1/\sqrt{\text {max}(m,n)}$ when $\lambda _{\text{ TV }}=0$. Otherwise, we recommend a smaller value for $\lambda$ because the total variation is also involved in the loss function for $\mathcal {S}_{(2)}$.

Our approach is unsupervised, that is, no expensive annotations of cells are required. Our model can be trained if there are multiple cytology images with sparse cells and redundant background. As shown in Fig. 2, our model can process cytology images in either color ($c=3$) or grayscale ($c=1$). Once trained, it extracts all the foreground cells in an input image and serves as an efficient background removal model. The encoder and decoder in the model compute cell features regardless of their sparsity, thus enabling the cell extraction even if the cells are nonsparse. The detection of cell regions can be achieved by identifying nonzero pixels in the predicted foreground image. Semantic segmentation can be readily accomplished by thresholding absolute pixel values.

## Experimental evaluation

### Background removal

**Fig. 3:**
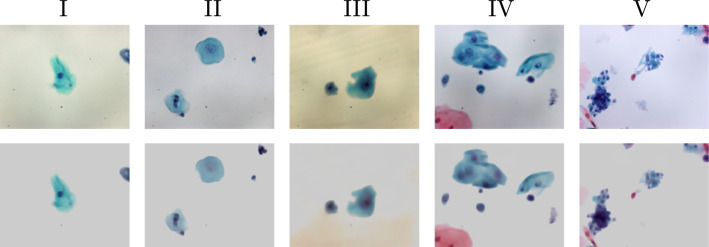
Examples of background removal test results on oral cytology images. The first row presents original images of Pap classes I to V. Our model predicts the foreground cells as the background removal results. Since the cells are darker than the background, their pixel values are negative in the prediction. To display the results as shown in the second row, we adjusted the predicted images by adding a uniform imaginary background of 0.8

We assessed the background removal of the Pap-stained cell images. The image dataset consisted of $5,\!041$ oral cytology images collected from 31 subjects at the Nagasaki University Hospital, as illustrated in Fig. 1. All color images were resized from $1,\!280\times 1,\!024$ to $320\times 256$ ($c=3$, $m=81,\!920$), and their pixel values were normalized to the range [0, 1]. We used twenty subjects to form a training dataset, six subjects for validation, and the remaining five subjects for testing. To train our model shown in Fig. 2 with the loss function in Eq. (1), we set the hyperparameters $\lambda =1/\sqrt{m}\approx 3.49\times 10^{-3}$ and $\lambda _{\text{ TV }}=5.0\times 10^{-3}$ and ran the Adam optimizer with a mini-batch size $n=32$ and a learning rate $\alpha =1.0\times 10^{-3}$ for 30 epochs. The background removal results for the validation images were insensitive to hyperparameters around these values. We observed stable convergence to the well-generalized model in this setting: the training loss almost monotonically decreased, and the validation loss did not tend to increase.

Figure 3 shows examples of the input and output of the model for the test images. Our model successfully predicted the foreground cells with the background removed in almost all the test images unless, as in the Pap class III example, the microscopic slide was heavily deteriorated. The small black spots isolated in the background are debris. Despite their sparsity, most of them were removed as well. This can be attributed to the total variation that makes the model learn to reject isolated pixels. As a side effect of the total variation, the predicted cells were slightly blurred.

### Debiasing cytology image classification

**Fig. 4:**
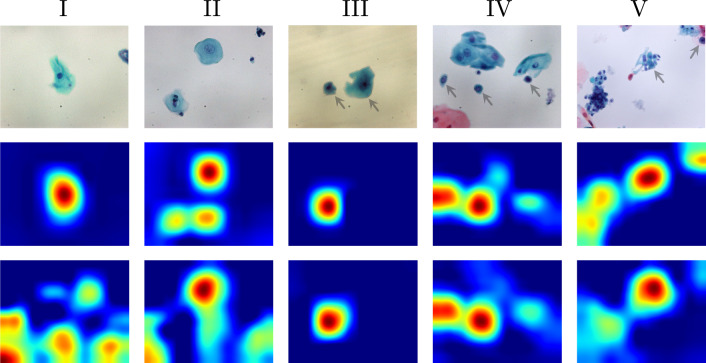
Distribution of class discriminative features with and without background removal. The first row shows an example of test image for each Pap class. The corresponding Grad-CAMs in the second row were obtained from a transfer learning classifier for the test images with background removed. The classifier was fine-tuned on oral cytology images with background removed. The first and third rows are the same as the first and second rows in Fig. 1

We confirmed the effectiveness of our background removal method in debiasing cell classification. We built an image classifier in the manner of transfer learning. We utilized the feature extractor of VGG16 [25], pre-trained on ImageNet [6], as the backbone of the classifier. A fully connected layer was attached to classify whether a cytology image was of normal (Pap class I or II) or abnormal (Pap class III or above) based on the global average pooling (GAP) [15] over 512 feature maps from the backbone. We trained a background removal model as described in the previous section and fine-tuned the classifier on the foreground cell images predicted by this model. We minimized the cross-entropy loss between the classifier outputs and the normal/abnormal labels using the Adam optimizer with a learning rate of $10^{-4}$ until the validation accuracy stabilized. For comparison, we also built an image classifier that was fine-tuned in the same manner as the original training images without using the background removal model.

We tested two image classifiers, one with background removal and one without, on test images. We present examples of Grad-CAMs [24] for each of the second and third rows of Fig. 4, respectively. Grad-CAMs highlight image features based on the inference of a classifier. The background-removed image classifier mainly focuses on the cells in all test image classes. Some of the expert-annotated cells of interest could be identified for the test images in Pap classes III–V (e.g., the cells indicated by arrows in Fig. 4). On the other hand, the classifier without background removal tended to infer on the basis of the background features for the test images of classes I and II in particular. It is difficult to find evidential features from cells to identify the cytology images of classes I and II as normal, compared to the images in classes III–V containing unique abnormal cells. It can be said that background removal hinders the extraction of discriminative features from patterns of background or debris that correlate with the subject.

We measured the performance of the two classifiers in identifying normal and abnormal cases by conducting repeated random subsampling ten times. The test accuracies with and without background removal were $76\%\pm 3\%$ and $81\%\pm 2\%$, respectively. These scores indicate that overestimation was corrected by addressing the background-bias issue, which is further explored in the Discussion section.

### Semantic segmentation performance

We extended the evaluation of our background removal approach in the context of a semantic segmentation task of cells. If our model can accurately subtract backgrounds from cytology images, only the foreground cells should remain. Therefore, we conducted quantitative evaluation of our model’s segmentation quality, utilizing an open dataset for cervical cell segmentation. We proved the superiority of our approach to supervised one and RPCA and assessed the effect of total variation.

#### Dataset and training settings

We evaluated the proposed semantic segmentation using the ISBI 2014 dataset [16, 17]. The ISBI 2014 dataset is intended as a benchmark for cell detection in cervical cancer cytology. The dataset consists of 135 synthetic grayscale cytology images of size $512\times 512$ ($c=1$, $m=512\times 512=262,\!144$) with segmentation annotation. We used only 45 images for training and validation purposes, whereas the remaining 90 images were used for testing according to the setup of the dataset. We evaluated our model ten times by cross-validation with repeated random subsampling, where $n=36$ out of 45 images were randomly selected for training each time. We normalized all pixel values to be in the range [0, 1] by simply dividing them by 255.

The hyperparameters including the binarization threshold were determined by Optuna [1] to maximize the Dice similarity coefficient (DSC) [7, 17] for the validation images. We found $\lambda \approx 1.14\times 10^{-3}$, $\lambda _{\text{ TV }}\approx 4.74\times 10^{-3}$, and the learning rate $\alpha =7.22\times 10^{-2}$ for the Adam optimizer [14]. The best binarization threshold was $6.9\times 10^{-4}$, which is much smaller than 1/255. We selected the best model for each round of cross-validation using the DSC.

**Fig. 5:**
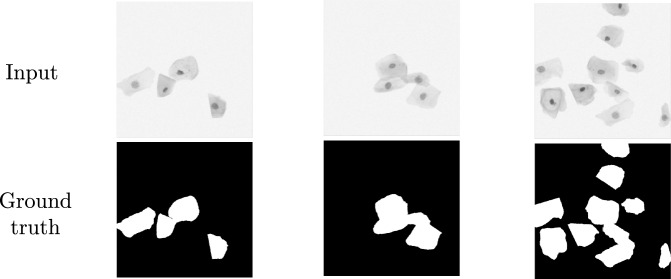
Examples of test images for semantic segmentation and corresponding ground truths in the ISBI 2014 dataset. Cells are sparse in the first and second examples, while they are nonsparse in the third example

**Fig. 6:**
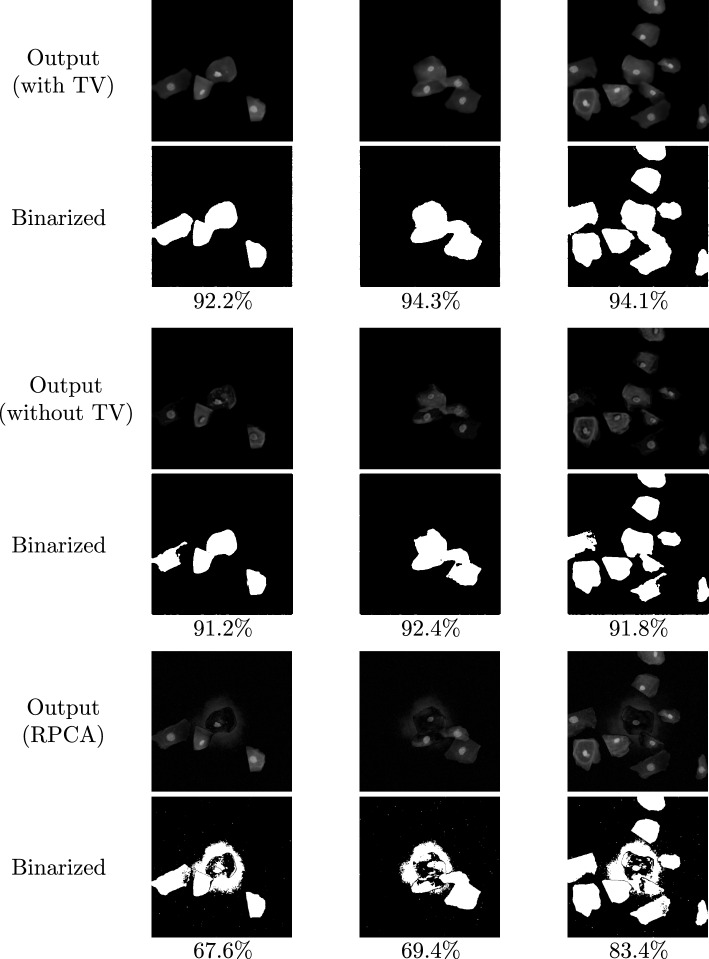
Examples of segmentation test results corresponding to the test images shown in Fig. 5. Output with TV shows the predicted foreground cells by our model, while the output without TV shows those by a dual-frame U-Net model trained without total variation for an ablation study. The output of RPCA shows absolute pixel values of sparse components, $\mathcal {S}$, computed by RPCA. The segmentation results are obtained by binarization of the outputs. The percentages indicate the Dice similarity coefficient (DSC)

#### Test results

Examples of the test images are shown in Fig. 5, and the corresponding model outputs are shown in the first and second rows of Fig. 6. The model successfully separated cells from the background. The binarization detects the cell regions with a mean DSC of $95.2\%\pm 5.5\%$ (mean ± standard deviation), which outperforms the algorithms presented in [17] and evaluated on the same ISBI 2014 dataset.

The segmentation performance was compared with that of a supervised model. We trained a dual-frame U-Net model with the same architecture as that in our model using the binary cross-entropy loss between its outputs and ground truths. The mean DSC of this supervised model for the test images was $58\%\pm 31\%$, which suggests overfitting to a small number of training images. We can conclude that our method offers a generalized model without supervision, even for small datasets.

#### Evaluation of TV effect

To evaluate the effectiveness of training with the total variation, we conducted an ablation study. We set $\lambda _{\text{ TV }}=0$ and revised the hyperparameters by Optuna as $\lambda \approx 1.05\times 10^{-3}$ and $\alpha =8.49\times 10^{-2}$. The best binarization threshold, $1.8\times 10^{-2}$, was much larger than 1/255, indicating a degradation in the ability to distinguish cells with nonzero pixels from the background.

The results of the cell separation and segmentation are presented in the third and fourth rows in Fig. 6. Some low-contrast parts of cytoplasm were missing or fragmented into tiny pieces. Incorporating the total variation in training was confirmed to encourage the formation of cell regions with connected pixels. Without the inclusion of the total variation, the mean DSC for the test images ranged between $74\% \pm 30\%$. Notably, the performance of the model was highly dependent on how the training data were split in the cross-validation. The total variation was found to stabilize the training, particularly in situations with limited data.

#### Comparison with RPCA

We compared our model with RPCA [4]. All 135 images in the ISBI 2014 dataset were reshaped into a single matrix $\textbf{D} \in \mathbb {R}^{135\times 262144}$. We employed the RPCA algorithm [10, 29] to minimize the objective function in Eq. (3) on the basis of the alternating directions method of multipliers (ADMM) [3, 9]. We implemented it using PyTorch [22] with double precision for fair comparison and numerical stability reasons. We have observed in preliminary experiments that the algorithm converges well within a thousand iterations. The optimal hyperparameter $\lambda$ was determined to be $\lambda \approx 1.1\times 10^{-3}$ using Optuna [1]. Optuna also found that the best binarization threshold was $4.8\times 10^{-2}$, significantly larger than that of our model.

The results of cell separation and segmentation by RPCA are presented in the last two rows of Fig. 6. RPCA failed to detect the cytoplasm of cells located around the center of the image and incorrectly detected background noise as foreground cells. Since most images in the ISBI 2014 dataset contained one or more cells in their center, portions of them were separated by RPCA into the low-rank backgrounds. The background noise was not of low rank; thus, it was identified as an outlier from plain background. Nonsparse cells, as in the third example, were almost detectable when RPCA was applied to the image of nonsparse cells along with sparse cell images. RPCA scored $71.9\%$ DSC for the test images, which was outperformed by our model thanks to the TV-sparse foreground feature learning with U-Net.

**Fig. 7:**
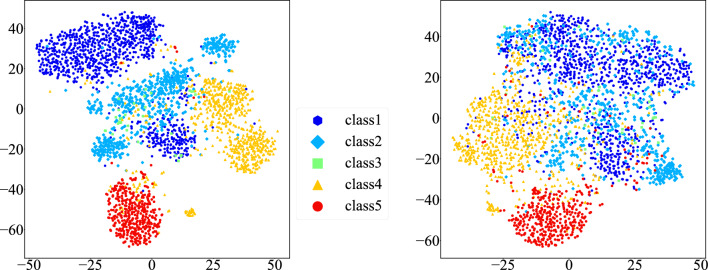
Comparison of GAP features distributions of oral cytology images with their original backgrounds (left) and backgrounds removed (right). The GAP feature vectors were obtained via a pre-trained VGG16 backbone and visualized by t-SNE. Each point corresponds to a cytology image, and the color represents the Pap smear class

Our model also outperformed RPCA in terms of computational efficiency. Our model can remove backgrounds from approximately 306 images per second at simple precision and 27 images per second at double precision using an A6000 GPU. Our model requires only one forward computation to process each test image, which is significantly faster compared to the iterative process involving singular value decomposition in RPCA until convergence. RPCA took $252\pm 2.3$ seconds to process 135 images, or approximately two seconds per image using the same GPU.

## Discussion

Grad-CAMs after the background removal and the decrease in the classification accuracy indicated that the background of cytology images contained discriminative features. Figure 7 shows the 512-dimensional GAP feature vectors extracted from oral cytology images by the feature extractor of the VGG16 pre-trained model before fine-tuning. In our oral cytology image dataset, the image features of Pap classes IV and V are separated from those of normal Pap classes I and II. However, except for well-separated class V, the Pap classes of the original images without background removal are composed of multiple clusters (left of Fig. 7). We confirmed that these clusters were mainly due to differences in background color. For example, Pap class I was divided into two clusters of images with white and yellow backgrounds owing to deterioration. Classification based on features that form clusters due to such background bias is clearly inappropriate. Removing the background from the images allows the classifier to extract image features independent of the background bias, resulting in each class not being composed of multiple clusters as shown on the right side of Fig. 7. The normal Pap classes I and II overlap widely, while the abnormal classes distinctive cell features, IV and V in particular, tend to be separated from the normal classes.

**Fig. 8:**
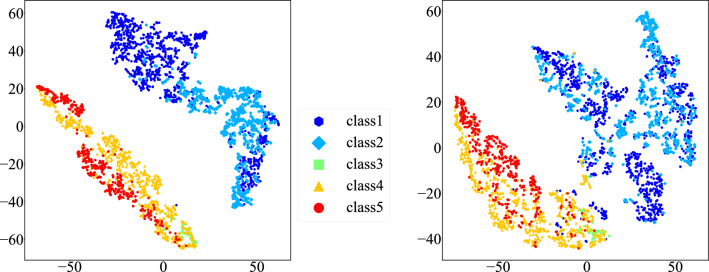
Same as Fig. 7, but the GAP feature vectors were obtained via VGG backbones fine-tuned on oral cytology images with and without their original backgrounds (left and right), respectively

Figure 8 depicts the t-SNE visualization of the GAP feature vectors extracted by the feature extractors of the transfer learning classifiers, which were fine-tuned on the original images (left) and the images with backgrounds removed (right). Both classifiers were fine-tuned to distinguish between normal and abnormal images. When background removal was applied, the abnormal Pap classes III–V were grouped away from the normal classes I and II. On the other hand, without background removal, the normal classes were grouped together excessively, while the clusters reflecting the background bias were still present.

**Fig. 9:**
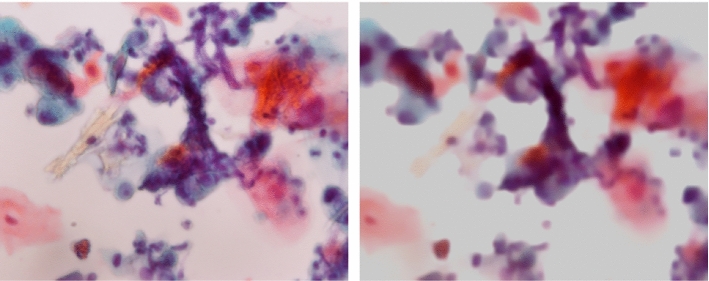
A cytology image of nonsparse cells (left) and that with background removal by our model (right)

Background-bias problems in deep learning have recently been addressed in computer vision applications such as video surveillance [27], object recognition in aerial images [28], and action recognition [8]. Typical solutions include background masking or foreground detection, bias elimination by random background replacement, and preprocessing to lower the ratio of the background. These solutions are designed for camera footage where the background is occluded by the foreground. For the background debiasing in cell diagnosis where the background is visible through the foreground cells, our proposed solution of cell and background separation is a reasonable approach.

Liquid-based cytology (LBC) has potential to be used in deep learning-based image processing. It prevents cell overlap and promotes cell sparsity, making it suitable for learning cell features. However, it should be noted that cell sparsity can lead to a relative increase in the background bias. Our solution applies to situations in which the training dataset comprises a batch of a few tens or more images with a low rank and sparse nature. In other words, the foreground cells are sparse and their backgrounds are similar. It is not necessary for the cells in the test images to be sparse. For instance, as shown in Fig. 9, it is possible to detect highly intricate cells (left) and replace the background (right).

Our unsupervised deep learning employs a loss function in Eq. (1) derived from RPCA, but it surpasses RPCA in several aspects. After training, our model can rapidly output foreground cell images via forward propagation. The total variation effect suppresses the detection of very small debris, and the foreground images output by the CNN appear natural and have few isolated sparse pixels. However, RPCA-based approaches have several disadvantages. The iterative method for minimizing the non-differentiable objective function incurs high computational costs when applied to a batch of training images. Furthermore, RPCA lacks generalizability: it is not designed to learn the features of the foreground cells of interest but the principal components of the backgrounds. The backgrounds learned from training images by RPCA are less informative for recognizing cells in test images.

## Conclusion

Cytology images may contain discriminative features in the background that should not be used for classification tasks. We showed that CNN classifiers had a high risk of learning background features because of the difficulty in finding cell features unique to Pap class I and II images. To avoid this background bias, we proposed an unsupervised deep learning model that separates cells from the background. Given a batch of cytology images that is sufficient to capture sparse cells against a redundant background, our model can learn the foreground cells without annotation. After training, our model detects cells even if they are not sparse and provides valuable images for classification without background bias.

## Footnotes

1 The oral cell images used in this article were collected at Nagasaki University Hospital.
