# Correction to: Background removal for debiasing computer-aided cytological diagnosis

**DOI:** 10.1007/s11548-024-03236-6

**Published:** 2024-08-09

**Authors:** Keita Takeda, Tomoya Sakai, Eiji Mitate

**Affiliations:** 1 School of Information and Data Sciences, Nagasaki University, 1-14 Bunkyo, Nagasaki, 8528521 Japan; 2 Department of Oral and Maxillofacial Surgery, Kanazawa Medical University, 1-1 Daigaku, Uchinada, Kahoku, Ishikawa 9200293 Japan; 3 Graduate School of Integrated Science and Technology, Nagasaki University, 1-14 Bunkyo, Nagasaki, 8528521 Japan

**Correction to: International Journal of Computer Assisted Radiology and Surgery** 10.1007/s11548-024-03169-0

In the original version of this article, received date 9 January 2024 was incorrect and should have been 20 March 2023.

The original article has been corrected.

